# Understanding the Biological Activities of Vitamin D in Type 1 Neurofibromatosis: New Insights into Disease Pathogenesis and Therapeutic Design

**DOI:** 10.3390/cancers12102965

**Published:** 2020-10-13

**Authors:** Claudia Riccardi, Lorena Perrone, Filomena Napolitano, Simone Sampaolo, Mariarosa Anna Beatrice Melone

**Affiliations:** 1 Department of Chemical Sciences, University of Naples Federico II, via Cintia 21, I-80126 Naples, Italy; claudia.riccardi@unicampania.it; 2 Department of Advanced Medical and Surgical Sciences, 2nd Division of Neurology, Center for Rare Diseases and InterUniversity Center for Research in Neurosciences, University of Campania Luigi Vanvitelli, via Sergio Pansini 5, I-80131 Naples, Italy; lorena.perrone@unicampania.it (L.P.); filomena.napolitano@unicampania.it (F.N.); simone.sampaolo@unicampania.it (S.S.); 3 Sbarro Institute for Cancer Research and Molecular Medicine, Department of Biology, Temple University, BioLife Building (015-00), 1900 North 12th Street, Philadelphia, PA 19122-6078, USA

**Keywords:** vitamin D, vitamin D supplementation, neurofibromatosis type 1, neurofibromin, therapeutics

## Abstract

**Simple Summary:**

We herein describe the relevance of Vitamin D for human health, with a special focus on its role in Neurofibromatosis type 1 (NF1) disease. Indeed, epidemiological studies revealed that low circulating vitamin D levels inversely correlate with cutaneous manifestations and bone abnormalities, clinical hallmarks of NF1. NF1 is an autosomal dominant syndrome with a severe predisposition in developing tumors and for which limited treatment options are thus far available. In this context, vitamin D or its analogues has been used to treat both skin and bone lesions in NF1 patients, alone or in association with other therapeutic agents. We provide an exhaustive and detailed analysis of the most relevant preclinical and clinical studies aimed at analyzing the correlation between vitamin D deficiency and NF1 lesion progression. This review can add a valuable contribution to the current knowledge of NF1 disease investigating possible therapeutic strategies to ameliorate NF1 conditions.

**Abstract:**

Vitamin D is a fat-soluble steroid hormone playing a pivotal role in calcium and phosphate homeostasis as well as in bone health. Vitamin D levels are not exclusively dependent on food intake. Indeed, the endogenous production—occurring in the skin and dependent on sun exposure—contributes to the majority amount of vitamin D present in the body. Since vitamin D receptors (VDRs) are ubiquitous and drive the expression of hundreds of genes, the interest in vitamin D has tremendously grown and its role in different diseases has been extensively studied. Several investigations indicated that vitamin D action extends far beyond bone health and calcium metabolism, showing broad effects on a variety of critical illnesses, including cancer, infections, cardiovascular and autoimmune diseases. Epidemiological studies indicated that low circulating vitamin D levels inversely correlate with cutaneous manifestations and bone abnormalities, clinical hallmarks of neurofibromatosis type 1 (NF1). NF1 is an autosomal dominant tumour predisposition syndrome causing significant pain and morbidity, for which limited treatment options are available. In this context, vitamin D or its analogues have been used to treat both skin and bone lesions in NF1 patients, alone or combined with other therapeutic agents. Here we provide an overview of vitamin D, its characteristic nutritional properties relevant for health benefits and its role in NF1 disorder. We focus on preclinical and clinical studies that demonstrated the clinical correlation between vitamin D status and NF1 disease, thus providing important insights into disease pathogenesis and new opportunities for targeted therapy.

## 1. Vitamin D: State of the Art

### 1.1. Vitamin D: Production and Metabolism

Vitamin D exerts fundamental biological functions in a variety of processes occurring in human tissues [1]. Sunlight exposure, dietary intake and supplementation represent the main sources of vitamin D. Once produced or ingested, vitamin D is subjected to a series of chemical transformations to produce the active form. The label vitamin D is a misnomer since it is not a vitamin but a fat-soluble, secosteroid hormone [2], which includes both vitamin D_2_, also known as ergocalciferol, and vitamin D_3_ or cholecalciferol [3].

**Figure 1:**
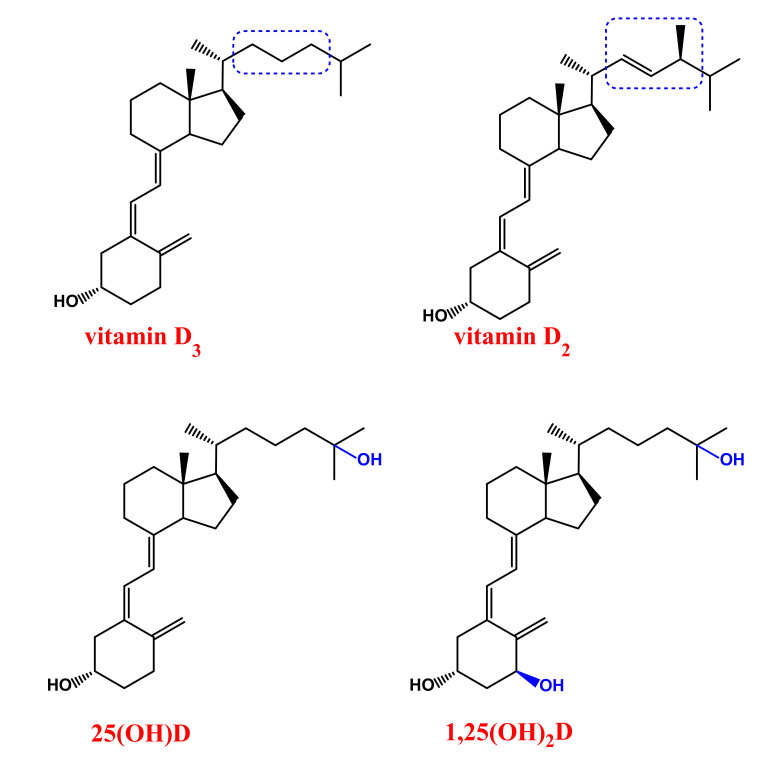
Chemical structures of vitamin D_3_, D_2_, 25(OH)D and 1,25(OH)_2_D. Structural differences are highlighted in blue. 25(OH)D: 25-hydroxy-vitamin D or calcidiol; 1,25(OH)_2_D: 1,25-dihydroxy-vitamin D or calcitriol.

From a structural point of view, vitamin D_2_ is a 28-carbon molecule, while vitamin D_3_ has a 27-carbon structure. Vitamin D_2_ differs from D_3_ also in the side chain composition, presenting a double bond between C-22 and C-23 and a methyl substituent on C-24 (Figure 1) [3].

**Figure 2:**
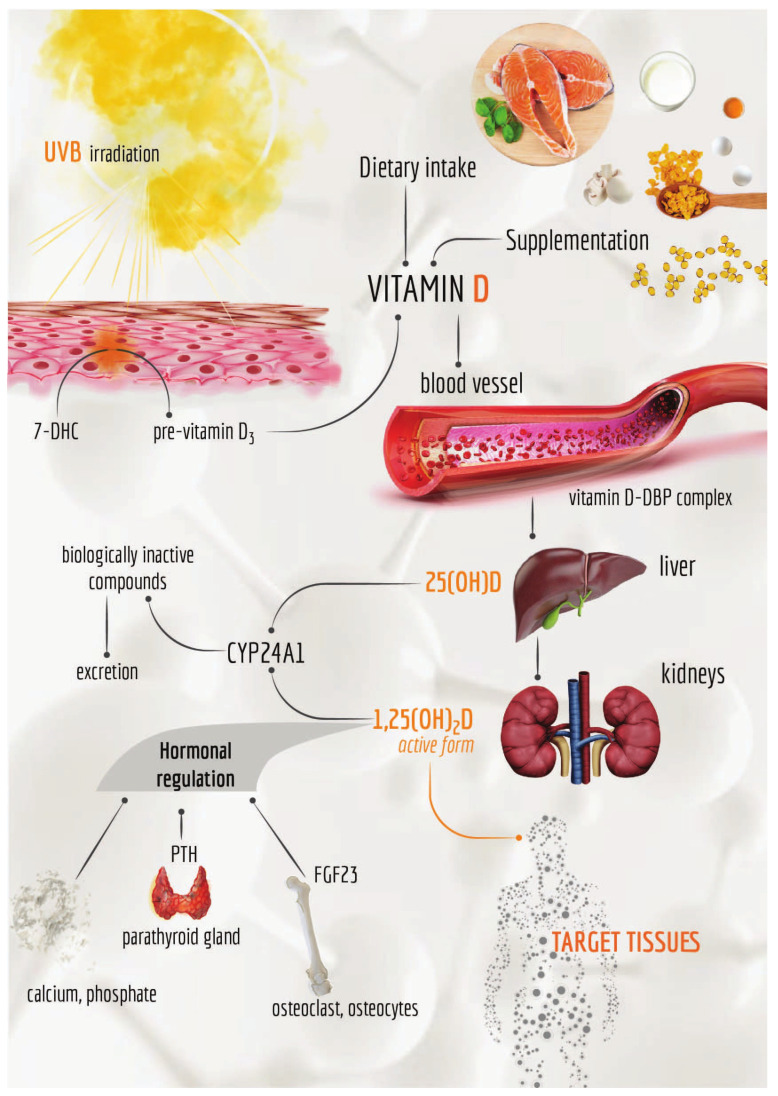
Schematic representation of the synthesis and metabolism of vitamin D. The main sources of vitamin D, i.e., supplementation, UV exposure and dietary intake are highlighted. A detailed description of the production of the active form, 1,25(OH)_2_D, and the regulation of its amount is also illustrated. UVB: ultraviolet B; 7-DHC: 7-dehydrocholesterol; DBP: vitamin D binding protein; PTH: parathyroid hormone; FGF23: fibroblast growth factor. Figure was redrawn from Szymczak-Pajor et al. [80].

Vitamin D_2_ body content almost entirely derives from the dietary intake—through plant source foods, mainly mushrooms—being produced from its precursor, ergosterol, by the action of ultraviolet B (UVB) radiation on plants, fungi, yeast and moulds [4]. On the contrary, only a small amount of vitamin D_3_ is obtained from the diet—primarily through animal source foods—while the major contribution derives from the endogenous synthesis starting from 7-dehydrocholesterol (7-DHC) substrate, which undergoes a series of photochemical and enzymatic conversions in the skin (Figure 2) [5,6]. 

In detail, UVB radiation (wavelength in the 290–315 nm range) penetrates uncovered skin providing energy for two subsequent non-enzymatically reactions, which occur in the plasma membranes of keratinocytes and fibroblasts [7,8,9,10,11,12]. Through the opening of its B ring, 7-DHC is first photo-converted in pre-vitamin D_3_, a thermodynamically unstable molecule, which is further thermo-isomerised to vitamin D_3_ via a slow temperature-dependent reaction (Figure 2) [9,10,12,13,14].

The amount of 7-DHC substrate available for the vitamin D_3_ production is indirectly controlled by the activity of 7-dehydrocholesterol reductase (DHCR7), an enzyme involved in the last step of cholesterol synthesis, which, in the case of the skin, competes with the vitamin D production since both pathways require the same precursor [15]. UV light intensity and the type of skin pigmentation also regulate the production of pre-vitamin D_3_ and, in turn, of vitamin D_3_ [16].

In the case of excessive exposure to UVB light, pre-vitamin D_3_ undergoes further photochemical reactions, involving the resealing of the B ring, thus producing a 7-DHC stereoisomer called lumisterol, or the isomerisation of the double bonds in the triene system to provide the compound named tachysterol [17]. The formation of both molecules is reversible, thus lumisterol and tachysterol can be converted back to pre-vitamin D_3_ when necessary [16]. The release of these biologically inactive compounds prevents the occurrence of vitamin D intoxication, which can be triggered by prolonged sun exposure [18].

Once produced, vitamin D_3_ can be released from the epidermal’ plasma membranes reaching the circulation system bound to the vitamin D binding protein (VDBP or simply DBP) [19,20,21], a member of the albuminoid superfamily produced in the liver [14,22]. Like albumin, DBP is a constitutive protein, able to transport vitamin D, whether produced in the skin or ingested, to the liver and kidneys [23], where it undergoes two sequential hydroxylation reactions, finally providing the biologically active metabolite.

In the liver, 25-hydroxylase enzyme catalyses the hydroxylation at C-25 of vitamin D, producing 25-hydroxy-vitamin D or calcidiol (25(OH)D, Figure 1), which is further hydroxylated—at carbon 1 of the A ring—by 1,α-hydroxylase to form the secosteroid hormone 1,25-dihydroxy-vitamin D or calcitriol (1,25(OH)_2_D, Figure 1) [23]. The enzyme 1,α-hydroxylase is mainly present in kidneys, where exerts its function within the cells of the proximal tubule. However, extra-renal sites of calcitriol production—such as bone, placenta, prostate, keratinocytes, monocytes, macrophages, T-lymphocytes and dendritic cells—have been also identified [18,24,25,26,27,28,29,30,31,32].

The structural differences between vitamin D_2_ and D_3_ are responsible for the reduced affinity of D_2_ for DBP, which results in a faster clearance of vitamin D_2_ and a lower conversion to 25(OH)D than vitamin D_3_ [33].

All the hydroxylase enzymes are cytochrome P450 mixed-function oxidases (CYPs) [34], located either in the endoplasmic reticulum (ER) or in the mitochondria [33]. The 25-hydroxylation reaction is mainly catalysed by CYP2R1 expressed in the ER of hepatocytes, with a possible contribution from CYP27A1 present in liver mitochondria [34,35,36,37,38,39,40], whereas CYP27B1 is the major 1,α-hydroxylase, exerting its activity mainly in kidney mitochondria [27,41].

Both 25(OH)D and 1,25(OH)_2_D metabolites are catabolised and thus inactivated by CYP24A1—an enzyme primarily localised in kidneys, catalysing the hydroxylation at C-24 and C-23 of calcidiol and calcitriol, respectively [33,42,43]. The products of these reactions are then oxidised, providing water-soluble products, ultimately excreted in bile and urine [23,44,45].

It is noteworthy to mention that in both liver and kidneys there are 3-epimerase enzymes, which—competing for the action on the same substrate—can inactivate all the vitamin D metabolites, producing analogues with the opposite configuration at only one stereogenic centre [46]. 

The overall production of 1,25(OH)_2_D is finely tuned by the serum concentration of calcium, phosphate, parathyroid hormone (PTH) and fibroblast growth factor (FGF23) [23,47]. Low calcium and phosphate amounts [48,49] promote CYP27B1 and suppress CYP24A1 activity, thus increasing the 1,25(OH)_2_D levels [16].

In the presence of low vitamin D status, PTH secretion—mediated by the parathyroid gland (Figure 2)—increases and promotes the activity of CYP27B1, leading to an enhanced conversion of 25(OH)D in 1,25(OH)_2_D [50,51,52,53,54]. In turn, circulating 1.25(OH)_2_D promotes the CYP24A1 activity while suppressing the CYP27B1 one; it also enhances the intestinal absorption of both calcium [55] and phosphate [15,47] and in parallel reduces the serum PTH levels by a negative feedback [56,57].

On the other hand, FGF23 is responsible for the phosphate metabolism, promoting the renal phosphate excretion by decreasing its reabsorption in the proximal tubule [58]. FGF23 is secreted by osteocytes in the presence of high 1,25(OH)_2_D concentrations: it induces the activity of CYP24A1 and inhibits that of CYP27B1, decreasing the levels of 1,25(OH)_2_D. At the same time, FGF23 can inhibit the production/secretion of PTH [59,60]. 

Therefore, 1,25(OH)_2_D represents the metabolically active form, involved in the regulation of calcium and phosphate homeostasis, antioxidant defense, inhibition of inflammation and cell proliferation [55,61,62,63]. 1,25(OH)_2_D can play different functions because of the tight autoregulation of its bioavailability through a series of negative and positive feedback mechanisms resulting in a fine-tuned expression of the hydroxylase enzymes, attending both metabolism and catabolism of the vitamin D [64].

In target tissues, upon dissociation from DBP, 1,25(OH)_2_D performs its main functions interacting with the vitamin D receptors (VDRs) [65], which, in turn, bind the retinoic acid X receptors (RXRs) [66], forming a VDR/RXR heterodimer. After nuclear translocation, the trimeric complex (calcitriol-VDR-RXR) binds specific DNA regions called “vitamin D responsive elements” (VDRE), which regulate the transcription of various genes. Other transcription elements can also bind this complex promoting either up- or down-regulation of the genetic activity [47,67,68,69,70].

Notably, VDRs are expressed in almost every tissue and organ such as the skin, placenta, bone marrow, brain, colon, breast, pancreas, thyroid, prostate, uterus, immune and malignant cells [3,16,71], and their widespread presence either in the cytosol or at the cell membrane is associated to the extra-skeletal effects of vitamin D [18,23].

The binding of 1,25(OH)_2_D to cytosolic VDRs is responsible of the genomic effects of vitamin D. On the other hand, in the nongenomic pathway, calcitriol binds to membrane bound VDRs (mVDRs), and 1,25D-membrane-associated, rapid response steroid-binding protein (1,25D-MARRS) receptors. Both interactions induce strong changes in cell signalling pathways through direct protein–protein interactions [70].

However, cutaneous formation of vitamin D_3_—through the described UVB-triggered process—is the most recognised natural source of this metabolite, accounting for 80–90% of vitamin D replenishment, thus labelling it as the ‘sunshine’ vitamin [14]. However, the dermal synthesis of vitamin D_3_ is modulated by numerous factors including photo-protective clothing and hats [72], skin pigmentation and melanin content, time of day, season, latitude [73], altitude and environmental aspects [11,18,23].

Modern lifestyle—including indoor living with insufficient sun exposure or the use of sunscreen [74,75] to prevent skin burns and cancer [76]—can significantly reduce endogenous vitamin D production [4,12]. For this reason, an adequate vitamin D intake through food or supplements became crucial [23,77,78,79].

### 1.2. Vitamin D: Dietary Intake, Supplementation and Toxicity

Vitamin D can also be exogenously obtained through dietary intake, which generally contributes only to 10% of the overall vitamin D levels [81,82]. Foods containing adequate levels of this micronutrient include cod liver oil and fatty fishes, such as swordfish, sardines, mackerel, salmon and tuna. Beef liver, egg yolks, yogurt and cheese represent additional natural sources of vitamin D, although contain it only in modest amounts [12,14,18]. Vitamin D in these foods is mainly present as D_3_ form [83], while some mushrooms provide the D_2_ form in variable amounts, the content of which is boosted by UV exposure [6]. 

Since only a few foods contain high levels of vitamin D, fortification of nutrients with this component represents a widely exploited strategy in various countries [84]. Products such as orange juice [85], margarine, milk and cereals are often fortified with vitamin D to prevent its deficiency [23,86].

Dietary vitamin D is absorbed from the gastrointestinal tract, predominantly in the small intestine via chylomicrons, which enter the lymphatic system [47]. The intestinal absorption ability may decrease in patients with hepatic, biliary, or gastrointestinal diseases [16,87]. 

Vitamin D supplementation represents the third and final source of vitamin D, available as over-the-counter products, for example, pills or oily drop preparations containing either vitamin D_3_ or D_2_ [47,88,89,90]. Vitamin D is thus incorporated into chylomicrons, which enter the bloodstream and bind both DBP and lipoproteins, allowing the conversion of vitamin D to the active metabolite through the above-described pathway [18]. A large body of experimental evidence suggested that vitamin D_3_ supplementation is, however, more effective than D_2_ in raising serum 25(OH)D levels [91,92,93,94,95]. 

The use of oral supplements is particularly advised for people at risk of vitamin D deficiency, i.e., elderly, breastfed infants and individuals with dark skin, living in countries with low sunlight exposure or suffering from malabsorption syndromes [18,23,96,97,98,99,100]. In Nordic countries, there is an evident variation in vitamin D status since during wintertime, in the absence of sufficient UVB light, serum concentrations of vitamin D markedly decrease in the population. 

Two clinical studies, i.e., VitDmet (NCT01479933) [101,102,103,104] and VitDbol (NCT02063334) [105,106], revealed a significant inter-individual variation in the average serum concentration of 25(OH)D and a personalised responsiveness to vitamin D_3_ supplementation, allowing to distinguish three different groups, i.e., high, mid and low responders. In detail, VitDmet investigated the long-term effects (for five months) of two daily low doses of vitamin D_3_ supplementation on glucose metabolism in pre-diabetic elderly individuals [101,102,103,104]. In contrast, VitDbol evaluated the effects of a single high-dose vitamin D_3_ oral bolus in young healthy adults already after one day [105,106]. Both studies unveiled that 25% of the participants could be classified as low responders [99] and thus more prone to develop vitamin D deficiency. Consequently, they need to increase their daily vitamin D_3_ doses compared to high responder people. Additionally, the observed variability in the vitamin D response index [107] suggested the necessity of a personalised vitamin D_3_ supplementation, rather than a general recommendation for the population [12].

However, while the prolonged sunlight exposure cannot produce toxic amounts of vitamin D_3_ in the human body because of the production of lumisterol and tachysterol, an excessive intake of vitamin D (through food or supplements) can represent a serious risk of hypervitaminosis D or vitamin D toxicity (VDT) [16,108,109]. Indeed, the widespread vitamin D fortification of foods and drinks from the 1930s to 1950s in the United States and Europe produced toxicity in certain individuals [110,111]. Major effects of vitamin D toxicity include increased calcium deposition in the body leading to hypercalcemia. Early symptoms of vitamin D toxicity include gastrointestinal disorders, for example, anorexia, diarrhoea, constipation, nausea and recurrent vomit. Additional symptoms are muscle and joint pain, kidney stones, apathy, drowsiness, continuous headaches, irregular heartbeat, loss of appetite, frequent urination, excessive thirst, weakness, nervousness and itching [111,112].

### 1.3. Vitamin D: Status, Measurement and Deficiency

The serum concentration of 25(OH)D is a well-established marker to assess the clinical vitamin D status in the human body [97,113,114,115]. The clinical advantages of choosing 25(OH)D instead of 1,25(OH)_2_D are numerous. First, 25(OH)D is the major circulating vitamin D metabolite and its levels remain stable for almost two weeks [31,116]. In contrast, 1,25(OH)_2_D has a short half-life of ca. 15 h and its serum concentration—closely regulated by PTH, FGF23, calcium and phosphate—does not appreciably decrease in the case of vitamin D deficiency [4,117].

Moreover, vitamin D toxicity proved to be associated with 25(OH)D concentrations [115]. Indeed, investigating the effect of vitamin D intoxication in two patients with normal kidney functions, Hughes et al. observed plasma 25(OH)D concentrations ca. 16-fold above the normal amounts, while 1,25(OH)_2_D levels were not significantly altered in both subjects [118].

Although there are controversial indications on the optimal vitamin D levels, serum 25(OH)D concentrations of <50 nmol/L (<20 ng/mL) are widely considered deficient, levels in the 50–80 nmol/L range (20–32 ng/mL) are indicated as insufficient and amounts ≥135 nmol/L (ca. 50 ng/mL) are generally accepted as sufficient [119]. 

Serum levels >250 nmol/L (>100 ng/mL) are associated with potential side effects [111,120]. However, optimal 25(OH)D concentration varies also according to age and sex [111], but these levels are generally used as reference ranges by most laboratories [120], in which the determination of total 25(OH)D amount is currently based on manual or automated radio-immunoassays and LC-MS/MS (liquid chromatography-tandem mass spectrometry) analysis [23,45,121]. Nevertheless, caution is warranted on the comparison between 25(OH)D values obtained in different laboratories or using distinct assays, since significant differences exist, requiring further standardisation of the current laboratory protocols [122,123].

Considering the synthetic pathway producing vitamin D, its deficiency may be a result of alteration in physiological ability such as hepatic or kidney dysfunction, decreased absorption, increased sequestration or catabolism [14,124,125]. Notably, mutations in the enzymes involved in metabolism/catabolism of vitamin D, i.e., DHCR7 [126], CYP2R1 [127], CYP27B1 [128], and CYP24A1 [42,43], have been associated with several diseases [15]. 

Additionally, environmental factors, for example, pollution or poor UVB exposure, as well as lifestyle, such as decreased outdoor activities and/or poor intake of vitamin D-rich foods, influence the aetiology of vitamin D deficiency [90,129,130]. 

Another common cause of vitamin D deficiency is medication use, for example, anticonvulsants or glucocorticoids, which can increase the catabolism of vitamin D [18,121].

In turn, vitamin D intake shows remarkable effects on the pharmacodynamics and pharmacokinetics of frequently used drugs, influencing their efficacy or promoting adverse reactions [131,132,133].

### 1.4. Correlations between Vitamin D Deficiency and Several Disorders

Vitamin D is essential for a series of physiological processes, such as the absorption of calcium and phosphate by the intestine, the regulation of bone remodelling and skeletal mineralisation as well as the negative feedback action on PTH secretion [3,98,134,135,136,137,138]. Therefore, in the case of a low vitamin D status, the capability of the small intestine to absorb dietary calcium [4,139,140] can be sensibly reduced with clinical implications not only for bone health but also for several metabolic functions [139,141]. 

A severe vitamin D deficiency influences the skeletomuscular system with bone mineralisation alterations, such as rickets [142,143,144] in children and osteomalacia [145,146] in adults. In particular, vitamin D-dependent rickets results in growth failure, hypocalcemia, elevated PTH levels and muscle weakness [147].

However, the presence of VDRs in almost every tissue and organ [3,16,71] suggests that vitamin D physiology extends well above and beyond its conventional role in calcium and bone homeostasis, highlighting its multiple biological effects [129,148,149,150].

In muscles, vitamin D maintains the integrity and improves muscle strength [151,152,153], while in kidneys, it reduces proteinuria and inhibits the renin-angiotensin-aldosterone system [154,155,156,157]. 

Interestingly, in diabetes, vitamin D supplementation promotes insulin secretion [80,141,158,159,160,161,162,163,164]. Indeed, 25(OH)D levels are typically lower in obese individuals who are more likely to develop diabetes mellitus and metabolic syndromes [165,166,167,168]. 

In elderly people, vitamin D status influences the occurrence of age-related diseases, such as age macular degeneration (AMD) [169,170].

Vitamin D plays an important role in reproduction, pregnancy, placental functions as well as foetal and child development, being involved in the prevention of preeclampsia, gestational diabetes and premature birth [171,172,173,174,175,176,177,178,179].

In the cardiovascular system, an adequate vitamin D status is associated with lower risks of hypertension and cardiovascular dysfunctions [180,181,182,183,184,185]. Optimal vitamin D levels are also important for the treatment and prevention of infectious diseases [186,187,188,189,190]. 

Additionally, vitamin D can modulate both adaptive and innate immunity [191]. Indeed, vitamin D deficiency is associated with several autoimmune diseases [192], for example, rheumatoid arthritis (RA) [193,194], inflammatory bowel disease (IBD) [195,196,197,198] and multiple sclerosis (MS) [199,200,201,202]. Associations between low vitamin D status and autoimmune thyroid diseases and thyroid cancer were also found [203].

Considering the high expression of VDRs in the brain [204], some studies investigated the correlation between vitamin D deficiency and the onset of neurodegenerative diseases [205,206,207,208], such as Alzheimer’s [209,210,211] and Parkinson’s diseases [212,213,214]. 

Since vitamin D also exhibits antiproliferative and pro-differentiating properties, its deficiency has been correlated with the progression of several malignancies [70,215,216,217,218,219], especially breast [220,221,222,223], ovarian [224,225] and skin tumours [226,227,228,229].

Noteworthy, the occurrence of low vitamin D levels has relevant implications also in the clinical manifestations of neurofibromatosis type 1 (NF1) [230]. We herein summarize the recent studies proving the interplay between vitamin D and NF1 disease and investigating the effect of vitamin D supplementation as an additive therapeutic strategy for NF1 patients.

## 2. Neurofibromatosis

The term neurofibromatosis includes at least three main distinct disorders, i.e., neurofibromatosis type 1 (NF1), neurofibromatosis type 2 (NF2) and schwannomatosis (SWN), sharing the propensity to develop multiple peripheral and central nervous system neoplasms. These are genetically determined, dominant hereditary disorders, each one featured by distinct genetic and aetiology grounds as well as peculiar clinical manifestations, as largely described in the literature [231,232,233,234,235,236,237,238].

These disorders, along with other critical illness, are collectively indicated as RASopathies, because of the crucial involvement of the Ras proto-oncogene in the appearance and progression of the disease [239,240,241,242].

NF1 (OMIM#162200), also known as von Recklinghausen disease, is the most common among these disorders and shows a complete penetrance, high variable expressivity and an estimated incidence of 1 in 2500–3500 live births, independently from the ethnic group, race and sex [231,243,244,245,246,247,248].

### 2.1. Genetics of NF1: NF1 Gene and Neurofibromin

NF1 is inherited in an autosomal dominant mode and is caused by mutations in the *NF1* gene, which is located on the long arm of chromosome 17, at q11.2. This gene spans over 350 kb of genomic DNA and comprises 59 constitutive exons and four alternatively spliced exons (9a, 10a-2, 23a and 48a) [249,250,251]. *NF1* gene encodes a cytosolic protein of 2818 amino acids, called neurofibromin, which acts as a tumour suppressor molecule [252,253].

Although molecular investigations in NF1 have been challenging due to the large size of the *NF1* gene, lack of mutational hot spots and the presence of pseudogenes, currently over 2600 different mutations have been reported in the Human Gene Mutation Database (HGMD), including missense, nonsense, deletions, insertions, intronic changes affecting splicing, alterations of the 3′ untranslated region of the *NF1* gene and complex rearrangements. *NF1* exhibits one of the highest spontaneous mutation rates in the human genome; indeed, almost half of the patients present de novo pathogenic variants [254,255,256,257]. The majority (>80%) of constitutional *NF1* mutations result in truncated forms of neurofibromin, which consequently shows loss of function or decreased activity [256,257,258,259]. The extreme clinical variability of NF1 disease suggests that random events intervene in determining the phenotype. Evidence in support of this interpretation is provided by the occurrence of somatic “second hit” mutation or loss of heterozygosity at the *NF1* locus that may influence the severity of the disease [260].

**Figure 3:**
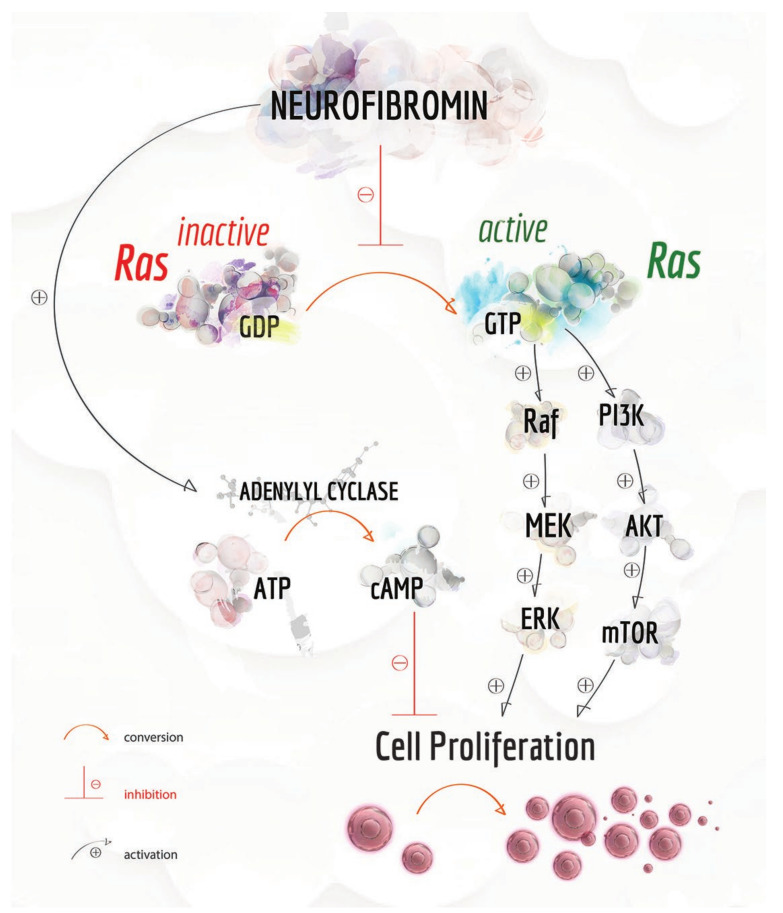
Schematic representation of the neurofibromin signalling pathways. Neurofibromin positively regulates adenylyl cyclase which, converting ATP in cAMP, increases the intracellular cAMP levels with consequent inhibition of cell proliferation. In addition, neurofibromin promotes the conversion of active GTP-bound Ras to its inactive GDP-bound conformation. The downstream effectors of the Ras pathway include mitogen-activated protein kinase (MAPK) and the phosphatidylinositol-3-phosphate kinase (PI3K). The activation of these Ras-triggered pathways leads to cell proliferation and tumour growth. Therefore, neurofibromin—acting as a negative modulator of the Ras signalling—exerts a tumour suppressor activity. Figure was redrawn from Lobbous et al. [283].

Although its expression levels vary on the tissue type, neurofibromin is ubiquitously expressed in multiple tissues, especially in the neurons and astrocytes of the central nervous system (CNS), as well as Schwann cells in the peripheral nervous system (PNS) [261,262], where the protein controls growth, survival, proliferation and differentiation of cells mainly via two intracellular pathways. In particular, neurofibromin negatively regulates Ras activity and positively control adenylyl cyclase (AC) functions. It is a GTPase-activating protein (GAP) and exerts its functions acting as a negative regulator of the p21ras (Ras) proto-oncogene, promoting its conversion from the active GTP-bound Ras to its inactive GDP-bound form (Figure 3) [263,264,265].

Active Ras promotes cell proliferation by activating several downstream signalling effectors including the mitogen-activated protein kinase (MAPK) and the phosphatidylinositol-3-phosphate kinase (PI3K), both associated with the NF1 phenotype. In detail, active Ras interacts with the serine/threonine kinase Raf, which, in turn, phosphorylates a second kinase, i.e., MAP kinase/ERK kinase (MEK). Then, triggered MEK promotes the activation of the Extracellular Signal-Regulated kinase (ERK) family. Once active, ERK phosphorylates a large variety of targets, regulating the expression of genes involved in the cell cycle, differentiation, migration and apoptosis. Besides the MAPK signalling cascade, Ras also activates PI3K, able to phosphorylate the protein kinase B (Akt or PKB). Akt activates the mammalian target of rapamycin (mTOR), which controls many cellular processes including cell proliferation and differentiation (Figure 3) [266,267,268,269,270,271].

Reduced or absent neurofibromin activity has been associated with the permanent activation of Ras (high Ras-GTP levels), leading to a constant stimulation of the Raf–MEK–ERK signalling cascade, which in turn promotes cell proliferation and tumour growth (Figure 3) [265,271,272,273,274,275,276,277,278].

Neurofibromin is also involved in a Ras-independent signalling pathway, being able to positively regulate the intracellular levels of cyclic adenosine monophosphate (cAMP). NF1 protein can activate the adenylyl cyclase, which converts ATP in cAMP (Figure 3), the primary function of which is the inhibition of cell proliferation [279].

Additionally, the MAPK pathway is negatively regulated by the SPRED (Sprouty-related protein with an EVH1 domain) family proteins, that are present in mammals as three homologs, i.e., Spred1, Spred2 and Spred3 [280]. It has been reported that Spred1 down-regulates the Ras/MAPK pathway through an interaction with the neurofibromin [281,282]. In turn, also the other Spred family members, Spred2 and Spred3, can interact with neurofibromin [281]. Importantly, this interaction functions to recruit neurofibromin to the plasma membrane, where it subsequently down-regulates the Ras-GTP levels. 

The ability of neurofibromin to control various molecular pathways relies on its peculiar functional domains [271,284,285]. The C-terminal region of the protein activates the adenylyl cyclase [279]. Conversely, the catalytic RasGAP activity of neurofibromin is performed by a centrally-positioned region, called GAP-related domain (GRD). GRD includes a central portion known as the minimal central catalytic domain (GAPc) and an extra region (GAPex) formed through the coiling of about 50 residues from the N- and C- extremities. The Ras-binding region is a shallow pocket located on the GAPc surface and is composed of highly conserved amino acid residues, known as arginine finger (residue 1276) [271,286]. Indeed, the transition state of the GTP to GDP hydrolysis is stabilised by the positively charged arginine finger, which neutralises the negative charges arising from GTP phosphoryl transfer, enhancing the catalytic ability of neurofibromin [287,288,289,290,291,292].

Notably, neurofibromin can be associated with microtubules via the GRD domain. For this reason, it is also indicated as a Microtubule-Associated Protein (MAP) [293,294].

Besides GRD, neurofibromin also contains a *Saccharomyces cerevisiae* phosphatidylinositol transfer protein (Sec14p homology-like) domain and a pleckstrin homology-like (PH) motif [285].

Sec14p is a lipid-binding domain found in secretory proteins and lipid-regulated proteins. This region is mainly composed of a lipid-binding pocket similar to a cage and is covered by a helical lid portion that controls the binding of ligands [271,295].

The PH-like moiety has a peculiar protrusion able to interact with the helical lid of the Sec14p domain. It seems that this connection controls the ligand access to the lipid-binding pocket, but its function has not been completely elucidated [296].

### 2.2. NF1 Protein Isoforms 

Alternative splicing of the *NF1* gene provides specific neurofibromin isoforms, which significantly vary in their sequence, tissue expression and capability to regulate intracellular pathways. In detail, five neurofibromin isoforms have been thus far identified, i.e., II, 3, 4, 9a and 10a-2 [271,297,298].

Neurofibromin type II, also indicated as GRD2 (domain II-related GAP) derives from the inclusion in the alternative splicing process of the exon 23a. This isoform is mainly expressed in Schwann cells and exhibits a low RasGAP ability. Neurofibromins of type 3 and 4 are both essentially expressed in muscle tissues. The first one (also termed 3′ALT) includes exon 48a, whereas the second one contains both 23a and 48a exons. In turn, the inclusion of the exon 9a in the alternative splicing process produces the isoform known as neurofibromin 9a or 9br, which shows limited neuronal expression and seems to play a role in memory and learning mechanisms. Finally, if exon 10a-2 is encoded, the produced isoform presents a transmembrane domain. This isoform is expressed in several human tissues, therefore it likely performs a housekeeping function in the intracellular membranes [271,299,300,301,302].

### 2.3. Clinical Manifestations of NF1 

Clinical manifestations of the NF1 disorder are widespread, involving the skin, bone and nervous system, unpredictable and variable, even within families with the same germline NF1 mutation [303,304].

**Figure 4:**
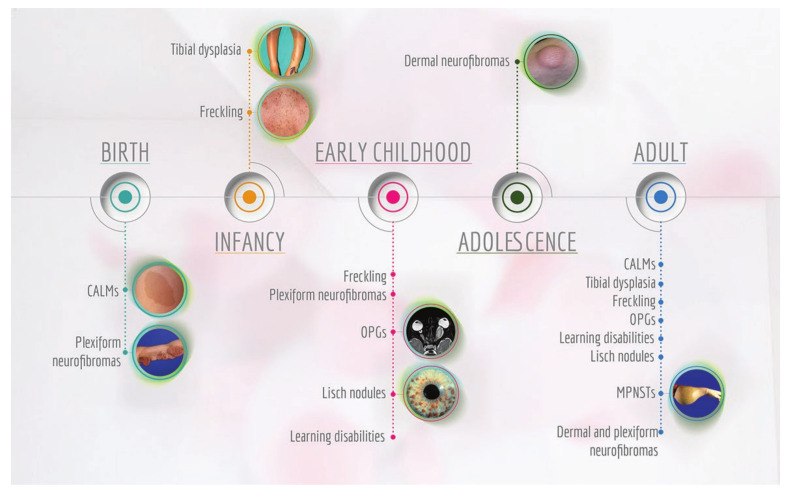
Main NF1 clinical manifestations arranged according to their corresponding age of onset. CALMs: café au lait macules; MPNSTs: malignant peripheral nerve sheath tumours; OPGs: optic pathway gliomas. Figure was redrawn from Williams et al. [246].

The bi-allelic inactivation of the *NF1* gene through a “second hit”—i.e., one allele constitutionally inactivated and the second somatically mutated—causes loss of heterozygosity of the *NF1* gene and seems to be important for the development of the disease manifestations [231,260,305]. Indeed, all the NF1 clinical manifestations are extremely variable and exhibit different and peculiar times of appearance (Figure 4) [246]. 

The primary phenotypic features of NF1 involve the skin with generalised skin hyperpigmentation, multiple café au lait spots or macules, pigmented freckling and neurofibromas [306].

Café au lait macules (CALMs) are benign pigmented lesions, flat, well-demarcated with homogeneous appearance. CALMs are typically indicated as “coast of California” when they show smooth borders or “coast of Maine” if rough borders are present. Typically, the colour is close to that of their namesake but can vary from tan to dark brown. CALMs get their pigment from melanocytes, which have an increased concentration of melanin. They usually appear in the first year of life and represent a common feature in all NF1 patients [246,307,308,309].

Skinfold freckling can be generally found in premature age of 3–5 years. These lesions are mainly axillary and inguinal but other common sites are the neck, trunk, breasts and around lips. Their size ranges from 1 to 3 mm, distinguishing them from CALMs, which are typically larger [310].

Neurofibromas—which can be cutaneous, subcutaneous and plexiform—are hallmark signs of the disease. Dermal neurofibromas are associated with a single peripheral nerve, while plexiform neurofibromas are associated with multiple nerve bundles. Cutaneous neurofibromas (CNFs) are mainly composed of fibroblasts, Schwann, mast and perineural-like cells. CNFs are dome-shaped dermal benign tumours with a soft and fleshy texture from flesh-coloured to slightly hyperpigmented. Conversely, subcutaneous tumours have a firm and nodular consistency [311].

Both cutaneous and subcutaneous neurofibromas may develop at any time of life, but their number and size are usually small before puberty and then increase after puberty and continue throughout adulthood [311]. Both types of neurofibromas generally do not transform into malignancy, but have a significant role in NF1 patients’ quality of life, causing both aesthetic and social problems due to their numbers and disfigurement [309,312].

Plexiform neurofibromas (PNFs) are pathognomonic of NF1 although it may occur in patients without other stigmata of NF1 [313,314]. PNFs have limited treatment options and can cause significant pain and morbidity: being highly vascularised, their complete surgical resection is very hard [313,315,316,317]. In contrast to cutaneous and subcutaneous neurofibromas, PNFs can become large and progress to malignancy forming malignant peripheral nerve sheath tumours (MPNSTs). These are aggressive soft tissue sarcomas, hard to detect and with a strong tendency to generate metastatic forms. MPNSTs are the most common cause of death for NF1 people because they can form in any nerve and do not respond to the current therapies [247,318,319].

In the evolution from PNFs to MPNSTs, a key form of transition is recognised in the atypical neurofibromatosis neoplasms of uncertain biological potential (ANNUBP). Compared to PNFs, ANNUBP show loss of neurofibroma architecture, high cellularity and high mitotic activity [320,321,322,323]. 

Other NF1-associated tumours are the optic pathway gliomas (OPGs), occurring in approximately 15% of NF1 individuals. They are most commonly found in young children (less than 7 years of age) and rarely in adolescents or adults [283,324,325,326,327]. These tumours may involve the optic nerve, chiasm and/or hypothalamus. Most are asymptomatic, but may interfere with vision or cause hypothalamic disturbance such as precocious puberty or other neurological symptoms [326,328,329].

Less frequent tumours found in NF1 patients are juvenile myelomonocytic leukaemia (JMML) [330], benign or malignant pheochromocytoma [331], gastrointestinal stromal tumour (GIST) [332], rhabdomyosarcoma [333], glomus tumours [334] and lipomas [335]. All the NF1-related tumours show bi-allelic inactivation of the *NF1* gene [336,337,338]. 

Ophthalmologic NF1 manifestations also include the so-called iris Lisch nodules (LNs), from the researcher who first observed them. These are melanocytic dome-shaped pigmented hamartomas of the iris varying in colouration from transparent to yellow or brown and usually appear in early childhood. Generally, a slit-lamp examination by an experienced ophthalmologist can reveal these asymptomatic changes in the majority of NF1 patients [303,310,316].

Extra-cutaneous manifestations of NF1 also involve focal or generalised bone abnormalities with dystrophic scoliosis, congenital pseudoarthrosis and bone dysplasia of tibia [339]. Tibial bowing occurs in an anterolateral direction and is usually visible in early childhood as bowing of the limb [340]. Generally, NF1 patients show mild short stature [341] and low bone mineral density (BMD) [342,343,344,345,346,347,348,349], making them prone to osteomalacia, osteopenia and osteoporosis [350,351].

It was also proved that NF1 patients have a reduced muscle force [352] and decreased bone strength [353] in comparison to healthy individuals. Indeed, bone architecture is strictly connected with muscle strength [354], which in turn plays an essential role in fracture risk. For this, bowing of tibia and fibula can also lead to pathologic fractures [355,356], especially in children and adults with 40 years or more [357].

Apart from skin and bone, NF1 has a major impact on the CNS [358]. Symptoms of attention deficit-hyperactivity disorder, mild cognitive impairment (including learning disabilities), delay in motor development, autism spectrum disorders and various problems in speech are common [242,303,359]. Patients with NF1 also exhibit symptoms of depression and anxiety, higher levels of perceived stress and lower levels of self-esteem compared with general population norms [360,361,362]. Less frequent in NF1 patients but, however, possible are vascular abnormalities such as the renal artery stenosis—with occlusion and stenosis of major intracranial vessels—or intracranial aneurysms [359,363,364,365,366,367,368].

### 2.4. Clinical Diagnosis of NF1

Although genetic tests are available [259,369], NF1 remains a clinical diagnosis and the majority of affected subjects are identified in infancy or childhood [370].

Developed by the National Institutes of Health [370], diagnostic criteria for NF1 are: 1. six or more café au lait macules with a diameter size of at least 5 mm in prepubertal patients and of 15 mm in postpubertal patients; 2. more than two axillary or inguinal freckles; 3. two or more cutaneous neurofibromas or 1 plexiform neurofibroma; 4. optic nerve glioma; 5. two or more iris Lisch nodules; 6. skeletal dysplasia or distinctive long bone abnormalities such as pseudoarthrosis; 7. first-degree relative affected by NF1 diagnosed according to the previous criteria. 

NF1 is diagnosed in an individual fulfilling two or more of these criteria. Approximately 95% of NF1 patients meet diagnostic criteria by age 8 and all of them do so by age 20. The diagnosis can be, however, difficult in patients who exhibit some dermatologic features of interest but who do not fully meet the diagnostic criteria [246,306,316,318,371]. 

In fact, some NF1 manifestations overlap with other RASopathies including Noonan syndrome, Noonan syndrome with multiple lentigines (i.e., LEOPARD syndrome) and Legius syndrome (LS) [241,372,373].

These clinically overlapping conditions make the unambiguous diagnosis of NF1 very challenging, which is very important for individualising clinical care and genetic counselling. However, specific strategies have been developed to identify genotype–phenotype correlations, demonstrating their full suitability to allow a differential diagnosis and guide the clinical follow-up of NF1 patients, especially when a clinical diagnosis cannot be established with certainty [374].

### 2.5. Treatment of NF1

Limited therapeutic options are so far available to treat NF1 clinical manifestations. 

Dermal neurofibromas can be removed by plastic surgery, use of the CO_2_ laser or electrodesiccation to improve their appearance and reduce their number [375].

For PNFs, their removal is often necessary to improve cosmesis or to reduce the pressure on the airway or spine. Unfortunately, PNFs are difficult to surgically remove due to their infiltration into adjacent normal tissue and after excision, regrowth frequently occurs [310].

Fluorodeoxyglucose positron emission tomography (FDG-PET) is a standard diagnostic approach to identify malignant regions and guide the biopsy [376].

Optic gliomas usually require chemotherapeutic treatment agents, such as vincristine or carboplatin [377]. MPNSTs are best treated by surgery and/or radiation therapy [319,323,378]. 

For the treatment of plexiform neurofibromas, clinical trials on different drugs, such as mirdametinib (NCT03962543), trametinib (NCT03741101) and binimetinib (NCT03231306), able to inhibit the Ras/MAPK pathway are underway in NF1 patients. Another MEK inhibitor, i.e., selumetinib, was granted the orphan drug designation by the Food and Drug Administration (FDA) for paediatric NF1 patients after very positive results of Phase II trials (NCT01362803) [379].

In turn, clinical studies on sirolimus (NCT00634270) and imatinib mesylate (NCT02177825, NCT01140360 and NCT01673009 [380]) have been completed.

A variety of mutation-directed therapeutics, which can be potentially used to treat NF1, are currently at different stages of clinical development [381].

Several studies reported the influence of diet and nutrition on the clinical features of NF1 disorder. Souza and colleagues indicated that NF1 patients consumed an unhealthy diet, rich in fats and sodium and poor in fibres and vitamins [382]. In another study, it was demonstrated that the use of a nutraceutical complex containing ginkgolide B/coenzyme Q10/riboflavin/magnesium can improve numerous clinical NF1 features, such as migraine-related disability [383]. Esposito et al. demonstrated that a Mediterranean diet and curcumin, a turmeric-derived polyphenol, induced a significant reduction in the number and size of neurofibromas, suggesting that an integrated and healthy nutritional supply can be effective in the management of NF1 subjects [384,385].

## 3. Vitamin D and Neurofibromatosis 1

### 3.1. Anticancer Effects of Vitamin D: Regulation of Multiple Signalling Networks

**Figure 5:**
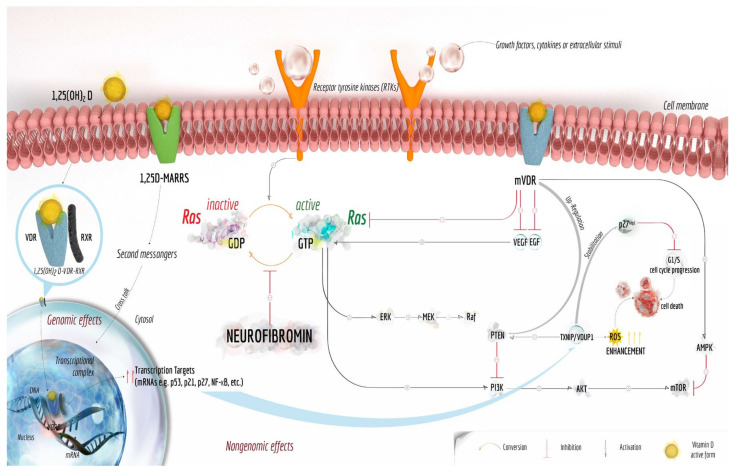
Multi-level regulation of vitamin D and integrated pathways with Ras-signalling controlled by neurofibromin. When 1,25(OH)_2_D binds VDRs present at the cell membrane, vitamin D influences different metabolic signalling molecules. Vitamin D inhibits the expression of both EGF and VEGF cytokines, as well as the Ras-signalling, while induces the activity of AMPK, which in turn, inhibits mTOR action. Conversely, upon binding to cytosolic VDRs, a trimeric complex with RXRs is formed, which then translocates in the nucleus and binds VDREs, finally regulating gene expression, including the transcription of VDUP1/TXNIP mRNA. In turn, VDUP1/TXNIP exerts several activities (L.P. is currently working on novel molecular pathways regulated from TXNIP): 1) enhancement of ROS with consequent extreme oxidative stress and cell death; 2) increase of activity of p27^kip1^, which inhibits G1/S cell cycle transition. Due to the action of TXNIP, p27kip1 is stabilised and retained in the nucleus where it exerts its inhibitory activity on the proliferation of malignant cells; 3) promotion of the active reduced form of PTEN, which inhibits the PI3K/ATK/mTOR signalling cascade. Vitamin D is also able to stimulate PTEN expression itself. On the other hand, neurofibromin promoting the inactive Ras-GDP form, completely inhibiting all the downstream effectors of the Ras-signalling cascade. RTKs: receptor tyrosine kinases; VDRs: vitamin D receptors; RXRs: retinoic acid X receptors; VDREs: vitamin D response elements; 1,25D-MARRS: 1,25D-membrane-associated, rapid response steroid-binding protein; EGF: endothelial growth factor; VEGF: vascular endothelial growth factor; AMPK: AMP-activated protein kinase; VDUP1: Vitamin D Up-regulated Protein 1; TXNIP: Thioredoxin Interacting Protein; PTEN: phosphatase and tensin homolog; ROS. reactive oxygen species.

The anticancer effects of vitamin D have been associated to both genomic and nongenomic effects of its active form, i.e., 1,25(OH)_2_D. In particular, upon binding to VDRs present at the cell membrane, vitamin D can limit tumour development influencing different metabolic signalling molecules by multiple mechanisms, including the regulation of growth factors (GFRs), mainly receptor tyrosine kinases (RTKs) (Figure 5) [386,387]. On the other hand, after binding to cytosolic VDRs, 1,25(OH)_2_D can induce immense changes in gene expression patterns in different cells, with several genes identified as either direct or indirect targets of the molecule [388,389].

Ben-Shoshan et al. reported the ability of vitamin D to inhibit the expression of both endothelial (EGF) and vascular endothelial (VEGF) growth factors [387,390], cytokines with key roles in cancer angiogenesis [391,392,393] and potent inductors of the Ras pathway activation [394].

On the other hand, besides directly inhibiting Ras-signalling [387], vitamin D induces AMP-activated protein kinase (AMPK), a pivotal intracellular energy sensor system that responds to energetic stress, for example, an increase in the AMP:ATP ratio or intracellular Ca^2+^ levels [395]. Once active, AMPK is able to directly inhibit the pro-survival downstream target mTOR (Figure 5) [396].

Among the putative vitamin D target genes is vitamin D Up-regulated Protein 1 (VDUP1), also called Thioredoxin Interacting Protein (TXNIP) because it has been isolated by different groups investigating distinct research areas: the genes responsive to vitamin D and the molecular pathways involved in oxidative stress response, respectively. Indeed, VDUP1 has been originally identified in HL-60 cells as one of the proteins, the gene of which is strongly induced by vitamin D (Figure 5) [397,398]. In turn, VDUP1 has been isolated in studies aimed to characterize the endogenous inhibitor of the reactive oxygen species (ROS) scavenger system of Thioredoxin (Trx) and for this, it was also called TXNIP [399] or Thioredoxin Binding Protein 2 (TBP2) [400].

VDUP1 plays a large variety of functions, from the regulation of cell proliferation to the induction of oxidative stress [398], as well as the promotion of inflammation and neurodegeneration [401,402,403,404,405]. Additionally, VDUP1 has been shown to act as an intracellular glucose sensor, responding to increases in glycolytic intermediates by limiting glucose uptake [386,389]. In turn, VDUP1 works to reduce intracellular glucose levels, by decreasing its uptake, possibly through limiting the membrane availability of glucose transporter 1 [389,406].

VDUP1 is necessary to preserve the activity of the tumour suppressor phosphatase and tensin homolog (PTEN), containing 2 cysteine residues (Cys71 and Cys124) that must remain reduced to exert its fundamental phosphatase activity. In detail, VDUP1 promotes the thioredoxin–NADP(H)–dependent reduction, favouring the active PTEN form with free thiol residues [407,408,409]. Additionally, vitamin D is able to stimulate PTEN expression itself (Figure 5) [410], which, in turn, inhibits the PI3K signalling pathway ultimately leading to mTOR activation (Figure 5) [409]. Therefore, both VDUP1 and neurofibromin, even if acting on different molecular effectors of the Ras-signalling pathway, behave as tumour suppressor agents blocking a key pathway that physiologically promotes cell proliferation. 

On the other hand, VDUP1 blocks the cell cycle and thus the cell proliferation by suppressing the activation of the promoter region of the gene coding for the cyclin A2 [411]. Because of its antiproliferative effects, VDUP1 is considered as a tumour suppressor [400]. In agreement, the expression of VDUP1 is frequently strongly down-regulated in a large variety of tumours, in which extensive methylation of the gene encoding VDUP1 occurs, resulting in a repression of its transcription [400]. Indeed, the transfection of cancer cells with a plasmid drives an enhanced expression of VDUP1 and leads to decreased cell proliferation [400]. Indeed, forced over-expression of VDUP1 in cancer cells decreases the translocation of the cyclin-dependent (CDK) inhibitor p27^kip1^ from the nucleus to the cytoplasm. Enhanced stability and presence of p27^kip1^ in the nucleus inhibits the CDK system of cyclin A, blocking the cell cycle transition from the G1 to the S phase and ultimately producing an arrest of the cell growth (Figure 5) [412]. Besides alterations in cell cycle regulation, tumour cells exhibit also elevated levels of ROS, leading to chronic oxidative stress (Figure 5) [413]. This sub-lethal increment of ROS participates in tumour progression by altering various signalling pathways [414]. However, in order to escape the lethal effect of ROS, cancer cells up-regulate the antioxidant systems [414,415].

Thus, therapeutic strategies aimed at enhancing the ROS levels at a lethal concentration are considered promising approaches to induce apoptosis of cancer cells. Noteworthy, a therapeutic strategy aimed at enhancing ROS levels by increasing the levels of VDUP1 has been shown against NF1-mutant malignancies—driven by excessive Ras signalling—as MPNSTs [416]. Indeed, combined treatment with mTOR and HDAC (Histone DeACetylases) inhibitors proved to kill NF1-mutant tumours of the nervous system both in vivo and in vitro [416]. This drug combination promoted catastrophic oxidative stress in MPNSTs by increasing the expression of VDUP1 and leading to a lethal ROS concentration [416]. Silencing of VDUP1 in NF1 tumours completely abolished cell death in response to mTOR and HDAC inhibitors, demonstrating that it was required in the induction of cell death following mTOR and HDAC inhibitors treatment.

These outcomes demonstrated that therapeutic approaches aimed at inducing VDUP1 expression can be effective against NF1 tumours [416]. Since VDUP1 is up-regulated by vitamin D, we may hypothesize that vitamin D may exert beneficial effects against NF1 tumours also by enhancing VDUP1 expression, opening the way to innovative therapeutic strategies in which vitamin D can be used in combination with other drugs, such as HDAC inhibitors, in order to increase VDUP1 expression, in turn promoting cell death of NF1 cancers.

### 3.2. Vitamin D-NF1 Correlation: Clinical Evidences

Several studies reported the strict association between vitamin D levels and cutaneous or bone manifestations in NF1 patients.

Lammert and colleagues enrolled 55 adults with NF1 and 58 healthy controls, both men and women from Germany with a mean age of ca. 40 years old [417]. Individuals with gastrointestinal, liver, kidneys, parathyroid glands or skin disorders—known to potentially affect vitamin D metabolism—were not included in this study and the same for individuals with unusually high sun exposure or using vitamin D supplements. Circulating 25(OH)D was evaluated in all the selected subjects in autumn and winter since vitamin D amount is known to be season-dependent [73]. Compared to healthy controls, the mean distribution of 25(OH)D concentrations was found much lower in NF1 individuals. In addition, an inverse correlation between the serum levels of vitamin D and the number of dermal neurofibromas was reported in NF1 subjects [417].

More recently, the role of the vitamin D receptor (VDR) was also explored by measuring its mRNA levels in 141 NF1 adult patients [418]. This study demonstrated that the number of dermal neurofibromas inversely correlated with both VDR mRNA and serum vitamin D levels in NF1 subjects, further corroborating the previous findings [417] and suggesting that low vitamin D content may contribute to the onset/development of the disease. On the contrary, PNFs and MPNSTs were not associated with these parameters [418].

Tucker et al. enrolled 72 adult NF1 individuals (29 men and 43 women)—excluding people with chronic illnesses or under treatments known to influence bone health—and 312 healthy individuals from Germany [356]. Then, serum vitamin D and PTH concentrations in both groups were measured in summer and winter. Most of the NF1 subjects showed 25(OH)D and PHT levels outside the standard reference range, respectively lower and higher than season-matched controls in both summer and winter. Additionally, NF1 subjects also showed low BMD consistently with osteopenia or osteoporosis. These findings were sex-dependent since males showed reduced BMD more likely than females. Pathological fractures were also reported only in NF1 individuals [356].

In Utah (USA), Stevenson and co-workers recruited 109 children with NF1 and 218 healthy subjects, with a mean age of 10 years. Children with NF1 included 59 males and 50 females and the control group was selected to match both age and sex parameters [419]. Similarly to previous studies [356,417], almost all NF1 individuals showed significantly reduced 25(OH)D levels than healthy individuals [419]. However, unlike previous observations [417], this study did not reveal a significant correlation between the reduction of vitamin D levels and the increase in the number of neurofibromas or optical gliomas in children with NF1 [419]. However, if we take due account of the age-related nature of neurofibroma formation, this relationship is more difficult to assess in paediatric subjects. In addition, differences in vitamin D levels observed in paediatric and adult NF1 population could be a consequence of geographical location or feeding habits [419].

Besides the vitamin D status, Hockett and colleagues also evaluated the muscle function (in terms of power, force and height of their jump) in children with NF1 with respect to their unaffected siblings coming from Germany [420]. A total of 30 children, 12 males and 18 females (aged 5–18 years), of which 15 with NF1 and 15 unaffected were enrolled. Thus, their serum 25(OH)D and PTH levels were measured. Children having any leg deformity, such as pseudoarthrosis of the tibia, fibula, or plexiform neurofibroma in the lower limb—known to affect leg length and mobility or the ability to jump—were not included in this study [420]. Differing from Stevenson et al. [419], this study revealed no significant variation in vitamin D status between NF1 children and healthy controls, observing in all cases low mean 25(OH)D concentrations. Conversely, the mean PTH concentration was significantly higher in children with NF1 compared to their unaffected siblings. In terms of muscular force, NF1 children showed impaired jumping power and force than healthy individuals [420].

In Canada, 18 children with NF1 were compared to their unaffected siblings in bone mineral content at the lumbar spine and proximal femur [421]. Subjects were selected between 6 and 20 years of age without focal bony lesions. Vitamin D and PTH levels were not significantly different between cases and controls, while NF1 subjects showed a sensibly reduced BMD compared to their healthy siblings. Additionally, affected children showed significantly lower bone strength, according to Hockett’s group results [420], corresponding to a higher lifetime fracture frequency [421].

To obtain more insights on vitamin D levels and metabolism in NF1 patients, Schnabel and co-workers evaluated 25(OH)D amounts in German children and adults with NF1 in winter and summer and compared the results with those obtained for healthy individuals. In detail, 58 adults and 46 children with NF1 were enrolled: adults had numerous dermal neurofibromas, whereas none of the children presented dermal NF1 manifestations [422]. This study generally confirmed low levels of vitamin D in NF1 adults compared to healthy subjects. In addition, vitamin D levels in NF1 patients were higher in summer than in winter, with a better trend found in affected individuals than in the control group, but without the levels found in NF1 patients reaching those in healthy adults, showing that simple sun exposure seems unlikely to account for the observed differences [422].

On the other hand, in the case of the paediatric population, there were no significant differences in the amounts of 25(OH)D between affected and healthy children in both seasons, with similar improvements from winter to summer for both the analysed groups [422], according to the results obtained by Hockett et al. [420], in German patients, but in contrast with those reported by Stevenson and colleagues [419] and Armstrong et al. [421], who analysed respectively American and Canadian patients. The observed inconsistencies may reflect a different dietary intake of this nutrient since food fortification of vitamin D represents a very common approach in North America, but not in Germany. These results corroborate the predominant role of the geographic position and sun exposure between people in USA and Germany, also suggesting an important connection to the age. Indeed, overall vitamin D levels were higher in healthy adults than in healthy children. Considering that children less frequently suffer from dermal neurofibromas compared to adults, vitamin D might act differently in younger patients. Particularly, the authors supposed a different age-related vitamin D metabolism [422]. 

In a recent investigation, Filopanti and colleagues proposed the trabecular bone score (TBS) as a tool for the measurement of bone microarchitecture and fracture risk in people with NF1 [423]. The authors determined vitamin D levels, vertebral and femoral BMD and TBS in 74 Italians with NF1 (26 males and 48 females) using a cohort of 61 voluntary healthy subjects (16 males and 45 females) as control group [423]. TBS was found lower in NF1 individuals without differences between sexes. As expected, 25(OH)D levels and BMD in hip and spine were also lower in NF1 subjects compared to healthy controls [423]. In the NF1 group, there was an evident association between serum vitamin D concentrations and the number of dermal neurofibromas, confirming previously reported data [417]. On the contrary, no correlations between TBS and 25(OH)D or the number of cutaneous neurofibromas were found [423].

In another study, along with the vitamin D levels, also *VDR FokI* and *BsmI* gene polymorphisms were examined [424]. Both *Fok*I and *Bsm*I polymorphisms can generate a decreased VDR expression [425,426,427,428], which in turn may reduce vitamin D effects, even in the presence of normal vitamin D levels. In 45 adults with NF1 (18–72 aged) from Southern Brazil, vitamin D amounts were measured and compared with those of 45 healthy controls matched by sex, skin type and age [424]. The differences in vitamin D levels between NF1 patients and healthy subjects were found not statistically significant, even if NF1 individuals showed reduced vitamin D levels. In particular, two patients with the lowest vitamin D levels also showed the largest number of cutaneous neurofibromas, corroborating the previous outcomes of Lammert et al. [417]. Moreover, a direct association between *VDR FokI* and *BsmI* gene polymorphisms and vitamin D levels in NF1 subjects was not observed suggesting that a low amount of 25(OH)D was not associated with these genetic variants [424].

### 3.3. Treatment of NF1: The Use of Vitamin D Alone or in Combination Therapy 

The unambiguous correlation between vitamin D levels and NF1 clinical features stimulated the use of vitamin D or its analogues as therapeutic agents as well as their combination with well-established Ras-pathway inhibitors for applications in NF1 manifestation treatment.

Nakayama and colleagues isolated primary fibroblasts from cutaneous neurofibromas of NF1 patients and demonstrated a remarkable cell growth reduction after treatment with vitamin D_3_ or its analogues, i.e., tacalcitol (1,24-dihydroxyvitamin D_3_) or 22-oxacalcitriol (22-oxa-1,25-dihydroxyvitamin D_3_), also known as OCT. However, different antiproliferative effects were observed on the selected fibroblasts as a consequence of the specific molecule tested [429].

Subsequently, fibroblasts, mast cells and Schwann cells were isolated from neurofibromas and their in vitro cellular growth was evaluated after vitamin D_3_ treatment and/or narrowband UVB (NB-UVB) irradiation [430]. The use of light irradiation (308 nm) sensibly reduced the proliferation of all the cell types, whereas the exposure to vitamin D_3_ or its synthetic analogue tacalcitol was effective only on the proliferation of fibroblasts and mast cells. A combination of calcitriol or tacalcitol with light irradiation provided additive effects on the cultured cells [430]. These results suggested that the response to vitamin D_3_ is cell specific and fibroblasts are the most sensitive cells [430].

On Schwann cells and fibroblasts isolated from neurofibromas, the same research group also examined the effect of rapamycin (or sirolimus), i.e., an mTOR inhibitor and lovastatin—a Ras-MEK pathway inhibitor—alone or in combination [431]. Schwann cells’ growth was reduced either by the use of rapamycin or lovastatin in a dose-dependent manner, whereas their combination resulted in additive inhibitory effect. Similar outcomes were also observed for fibroblasts although with effect slightly lower than those reported in Schwann cells. A combination of vitamin D_3_ with rapamycin and/or lovastatin was also explored: the use of calcitriol slightly strengthened the efficacy of either drug in Schwann cells, while in fibroblasts additive effects were found [431].

In a different study, neurofibroma tissue was transplanted subcutaneously into nude mice skin and OCT was administered. With respect to the untreated mice, the growth and the density of neurofibroma tissue was found to be sensibly reduced by daily, local OCT injection [432]. Subsequently, the topical application of OCT to nude mouse skin for six months proved to be effective in reducing the pigmentation of café au lait spots [433].

Stimulated by these intriguing in vitro and in vivo findings, the same research group also investigated the effects of NB-UVB irradiation on the serum vitamin D levels in NF1 patients [434]. Nine subjects were enrolled, including two men and seven women with an age ranging between 21 and 81 years (mean age of ca. 42 years) [434]. Once weekly or biweekly, NB-UVB irradiation proved to markedly increase the serum 25(OH)D levels, with detectable differences after 18 months between treated and untreated groups [434]. Time-course analyses of the serum 25(OH)D levels in the treated NF1 patients revealed that the overall concentrations became significantly higher after six months of irradiation, then reaching a plateau since the prolonged treatment did not provide any additional beneficial effect [434].

The same research group also evaluated the efficacy of intense pulsed-radiofrequency (IPL–RF) combined with the topical application of OCT ointment (Maxacalcitol, OXAROL®, Chugai Pharmaceutical, Tokyo) for the treatment of NF1 pigmented lesions [435]. Indeed, IPL–RF was proved to be absorbed by melanin pigments producing their direct destruction [436]. First, the authors reported a single case of a 27-year-old woman successfully treated over four months with this combination therapy: indeed, an increased lightness of her skin was detected [437]. Thus, the authors extended the investigation to eight NF1 patients (two males and six females) aged 3–38 years (with a mean age of 20 years), which were treated for almost two years in different body sites such as face, neck, trunk and legs. IPL–RF/OCT combination improved the appearance and reduced the number of small pigmented freckling more than CALMs, with a moderate to good response in six of the eight treated patients [435]. Furthermore, no topical or local anaesthesia was used during IPL–RF exposure and no re-pigmentation was observed for the successive six months after treatment [435]. Thus, IPL–RF proved to be remarkably more advantageous compared to other laser-based approaches explored for cutaneous manifestations, which need local anaesthesia and sometimes resulted in skin scarring or showed a high rate of recurrence [438].

Since supplementation with calcium and vitamin D_3_ demonstrated beneficial effects on bone mineral density, this combination was also evaluated in NF1 patients with general bone abnormalities [439,440].

Brunetti-Pierri et al. investigated bone status in 73 NF1 subjects, 26 males and 47 females, mainly children and adolescents (mean age of ca. 16 years) [350]. In a subgroup of 16 subjects with marked osteoporosis and osteopenia, they found a statistically significant and generalised reduction in bone mass compared to normal controls. Additionally, in this subgroup, 8 individuals also showed slightly higher serum PTH concentrations and 10 patients had a serious vitamin D insufficiency. These subjects were specifically selected to measure bone turnover and bone density before and after vitamin D_3_ and calcium treatment [350]. After four months of supplemental therapy, PTH was increased to normal levels in 6/8 subjects, but lumbar spine BMD did not significantly change over two years [350]. In contrast, subsequent investigations proved the combination of vitamin D_3_ and calcium beneficial for BMD improvement.

Seitz and colleagues examined 14 adults (five men and nine women) affected by NF1, aged in the 19–66 years range, with an average age of ca. 44 years [441]. To avoid external influences on the bone turnover, individuals with secondary pathologies such as primary hyperparathyroidism, hyperthyroidism, renal or hepatic disorders, malabsorption syndrome, or rheumatic diseases were excluded from this study [441]. The control group included 15 males and 27 females with a mean age of 47 years. For NF1 subjects, histologic analysis of iliac crest biopsies revealed an increased osteoid volume associated with a higher number of osteoblasts and osteoclasts compared to biopsies from healthy individuals. Additionally, NF1 patients showed significantly lower 25(OH)D serum levels and decreased BMD with respect to healthy controls, accompanied by high PTH levels. Hence, a combination of vitamin D_3_ and calcium was administered for one year in a subgroup of 4 patients with remarkable reduced BMD [441]. After this treatment, both vitamin D and PTH serum levels were normalised and a significant improvement in BMD in the spine but not in the hip was observed [441].

Similar outcomes were also achieved by Schnabel and colleagues, who focused on the effects on the hip and lumbar spine of adult NF1 patients with vitamin D_3_ deficiency [442]. The serum levels of 25(OH)D and BMD were determined in 35 adult subjects with NF1 (12 men and 23 women, with age ranging from 32 to 63 years). 19 patients received vitamin D_3_ supplementation for two years, 6 patients for one year and 10 patients no received supplementation [442]. Compared to untreated individuals, treated subjects showed a significantly improved BMD especially at the level of the hip [442].

The evaluation of bone mineral metabolism parameters in NF1 patients, before and after calcium and vitamin D_3_ supplementation, was also performed by Petramala and colleagues [339]. The authors evaluated 70 adult NF1 patients (37 men and 33 women, mean age ca. 40 years) and 40 normal subjects (22 men and 18 women with a mean age of ca. 44 years). Individuals affected by renal failure, cardiovascular or thyroid dysfunctions were excluded from this study [339]. A total of 35% of NF1 patients showed bone alterations featured by reduced BMD of the lumbar spine and femoral neck associated with an increased prevalence of osteopenia or osteoporosis. Moreover, NF1 individuals exhibited severe hypovitaminosis D and high PTH levels than the control group [339]. For the first time, reduced magnesium levels were reported in NF1 patients: magnesium is important for bone health since its reduction can promote the development of reduced bone mass [339]. After one year of supplementation of calcium and vitamin D_3_, a significant increase in 25(OH)D and magnesium levels, a sensible reduction pf PTH levels and general improvements in the bone mass were observed [339].

In a subsequent study, 6 patients with NF1-related osteoporosis were enrolled to evaluate the efficacy of vitamin D_3_ treatment in combination with alendronate [443]. Alendronate is a bisphosphonate medication able to target osteoclasts and inhibit farnesyl diphosphate synthase, interfering with the farnesylation of small GTPases including Ras, Rac and Rho [444,445]. This process leads to osteoclast apoptosis, an increase in BMD and a reduction in fracture risk [446,447].

Alendronate is used for the prevention and treatment of osteoporosis [448]. In this study, for almost two years, a weekly dose of alendronate and a daily vitamin D_3_ supplementation was administrated to five men and one woman, aged 28–76 years [443]. After the treatment, BMD was increased in five out of six patients, but this increase was not statistically significant. A new stress fracture of the tibia was also documented in a patient under therapy. Unfortunately, this investigation did not provide unambiguous results on the effects of vitamin D_3_/ alendronate combination on NF1-related osteoporosis patients because of the low number of enrolled individuals [443]. 

Regarding the clinical trials, the U.S. National Institutes of Health reports only one vitamin D supplementation study in NF1 patients (searching for “neurofibromatosis” and “vitamin D”). The aim of this investigation (NCT01968590) was to evaluate the treatment of adult NF1 patients (25–40 years old), who show insufficient serum 25(OH)D levels, with two different doses of vitamin D supplementation over two years and to verify if improvement in the BMD loss can be achieved over this time. To the best of our knowledge, the results of this study are not yet available.4. Conclusions and Perspectives

The complexity of the large *NF1* gene and its several variants without definitive genotype–phenotype correlations represent a strong challenge in NF1 treatment for both clinicians and researchers. Undoubtedly, in the past two decades, outstanding progress has been made in the understanding of both the pathophysiology and genetics of NF1 disease. Management of this disorder currently consists of surveillance, surgical treatment of progressive lesions and genetic counselling. 

Although some suitable animal models have been developed, NF1 manifestations still lack proper model systems to be clinically and molecularly investigated. Thus, effective and definitive therapeutic modalities have not been established yet, but several trials are ongoing to discover and test valuable treatments for the various cutaneous and non-cutaneous manifestations of the NF1 disorder.

The metabolism of vitamin D—regulating a broad spectrum of physiological processes—proved to play a key role in the pathogenesis of NF1 disease. Thus, personalised nutrition including a tailored vitamin D supplementation may represent a very useful approach in the maintenance of wellbeing of NF1 patients and their management.

In NF1 individuals, vitamin D deficiency can worsen bone metabolism, promoting reduced bone mass and general bone abnormalities. Thus, the restoration of healthy vitamin D status can be an effective therapeutic intervention in NF1 patients.

Vitamin D levels also affect NF1 cutaneous manifestations, especially CALMs and neurofibromas. Several molecular mechanisms could be at the base of the association between low serum vitamin D levels and the occurrence of neurofibromas in NF1 subjects.

Individuals with a huge number of dermal neurofibromas can be more prone to cover their skin for aesthetic embarrassment or discomfort and decrease their outdoor activities, especially in the presence of associated NF1 comorbidities (e.g., scoliosis, pseudoarthrosis).

Clothing selection and lifestyle habits of NF1 patients can affect the sunlight irradiation usually received, or conversely, the increased pigmentation of their skin negatively influences the efficiency to produce an adequate amount of vitamin D, even in the presence of adequate light exposure. 

To better understand the vitamin D levels in NF1 patients, studies on vitamin D intake, absorption, synthesis, transport, or catabolism in affected individuals could be very useful.

It would also be interesting to explore a large variety of dietary supplementation based on the combination of vitamin D with other healthful agents—for example, polyphenols, known to exert neuroprotective functions [449,450,451]—with the aim to test possible synergistic effects.

Moreover, vitamin D has also been incorporated—using calcitriol or some of its analogues as active components—in different drug delivery systems for food fortification or therapeutic applications [452,453,454]. To the best of our knowledge, these micro/nano-formulations have not been tested in NF1 patients; so this strategy can be attempted in the next future to improve NF1 patient management and their life quality.

As NF1 remains a multisystem disease with life-threatening complications, a multidisciplinary approach with close collaboration among clinicians and researchers will be needed for the diagnosis and management of this condition.

Efforts at standardising the outcome assessment in NF1 clinical trials could be very useful to add reliable data for definitive disease treatment. Also the role of the microenvironment and the patient characteristics such as sex, which influence the vitamin D status, should be further investigated in NF1 patients, being a key factor that can contribute to a precision medicine-based approach to fight NF1 disorder.
